# Multiparametric Magnetic Resonance Imaging in Evaluation of Benign and Malignant Breast Masses with Pathological Correlation

**DOI:** 10.7759/cureus.22348

**Published:** 2022-02-17

**Authors:** Varshitha GR, Anil K Sakalecha, Asadulla Baig

**Affiliations:** 1 Radiodiagnosis, Sri Devaraj Urs Medical College, R.L. Jalappa Hospital, Kolar, IND; 2 Department of Surgery, Sri Devaraj Urs Medical College, R.L. Jalappa Hospital, Kolar, IND

**Keywords:** brest cancer, kinetic curve, adc value, diffusion weighted imaging (dwi), mr mammography

## Abstract

Background

Dynamic contrast-enhanced (DCE) MRI sequences plays a vital role in diagnosing breast masses with high sensitivity and specificity as compared to other diagnostic modalities. The addition of diffusion-weighted imaging (DWI) and apparent diffusion coefficient (ADC) values significantly improves diagnostic accuracy. This study aimed to study the breast masses on DCE-MRI, restricted diffusion on DWI, ADC values, and choline peak on spectroscopy in breast cancer diagnosis.

Material and methods

This study was a prospective observational study which involved subjects with breast lumps. Baseline data was collected from the patients along with pertinent clinical history and relevant laboratory investigations. MR mammography (MRM) was performed on a 1.5 Tesla MR Scanner (MAGNETOM® Avanto, Siemens AG, Munich Germany) using a dedicated double breast coil.

Results

Forty-one subjects were included with a total of 54 breast masses in them. The mean age of the study population was 47.1±14.7 years. From the MRI final diagnosis, the majority (53.70%) were diagnosed as malignant lesions and 46.30% as benign. Out of 20 lesions diagnosed as benign on histopathology, only five percent had ADC value <1.3 ×10^−3^mm^2^/s, and the majority (95%) had ADC value >1.3 ×10^−3^mm^2^/s. All 20 lesions were circumscribed, ovoid, or round in shape showing no restricted diffusion on DWI, with corresponding ADC value of >1.3×10^−3^mm^2^/s, homogeneous post-contrast enhancement, or with dark internal septations, type I kinetic enhancement curve, and they showed no choline peak on spectroscopy. Out of 34 malignant lesions diagnosed on histopathology, the majority (85.29%) displayed restricted diffusion on DWI and had an ADC value of <1.3×10^−3^mm^2^/s, most of them had spiculated margins, type II/ III kinetic curve with choline peak on spectroscopy.

Conclusion

Multiparametric MR mammography, which included DCE-MRM, DWI, ADC values, and spectroscopy, correlated well with the histopathological diagnosis of benign and malignant breast masses.

## Introduction

Breast cancer is the most frequent cancer diagnosed in women and is the leading cause of death among females [1]. It is a multifactorial ailment, and several factors contribute to its incidence. Even though the disease occurs globally, its frequency, death rate, and survival rate differ noticeably among various parts of the world. This may be due to the type of population, genetic factors, and demography [2]. Variations in risk factors have led to an upsurge in the frequency of breast cancer, which is growing every day.

According to the World Health Organization (WHO), the prevalence of breast cancer in women globally is 2.3 million in 2020, and mortality was found in 685,000. The death rate in breast cancer is mainly due to extensive metastasis. From the last five-year data up to 2020, there have been nearly 7.8 million new cases diagnosed with breast cancer and therefore, making it the most dominant cancer globally. Breast cancer can occur at any age post-puberty; however, the incidence is greater at an older age. The mortality rate has decreased over the years due to the early diagnosis by mammography, sonomammography, MRI, and early intervention [3].

MRI is an emerging modality in detecting and characterizing breast lesions. It is accurate in detecting lesions within the dense breast, subcentimetric lesions, and those not conclusive on X-ray mammography and sonomammography. MRM has a 90-100% sensitivity and 85-90% specificity in detecting breast carcinoma [4]. The reason behind the increasing adoption of MR mammography (MRM) at many hospitals is its intense sensitivity for the detection of breast cancer. Several studies showed that contrast-enhanced (CE)-MRM has a high sensitivity of all other imaging modalities in asymptomatically high-risk women and in patients having breast cancer which was recently diagnosed [5,6].

Dynamic contrast-enhanced (DCE)-MRI is the mainstay of MRI protocol. It is the subtlest method used in the diagnosis of breast cancer [7]. Its high resolution with good morphological data and information on neo-angiogenesis makes it one of the best methods for tumor detection [8]. Common indications for CE-MRM currently include supplemental screening for high-risk female patients, preoperative assessment of the extent of breast cancer, evaluation of equivocal findings on other preliminary imaging and/or clinical examination, and evaluation of cancer response to neoadjuvant chemotherapy [9]. Multiparametric MRI (mpMRI) protocols include diffusion-weighted imaging (DWI), apparent diffusion coefficient (ADC), and MR spectroscopy, along with DCE-MRI. It can also assess regional lymph nodes which have metastasized [10]. These sequences increase the sensitivity and specificity of DCE-MRI and thereby provide better imaging diagnosis.

Therefore, additional approaches to improve the specificity of DCE-MRI in this patient cohort are essential. Therefore, the intention of this particular study was to evaluate the use of mpMRI in assessing breast lesions. To derive sensitivity and specificity of mpMRI as compared to histopathology in diagnosing benign and malignant breast lesions. We also assessed the role of DWI sequence in assessing benign and malignant axillary lymph nodes and derived their cutoff ADC value.

Components of the multiparametric breast MRI protocol

MRM has advanced from a primarily contrast-enhanced method to a multiparametric method, in which T1-weighted (T1W), T2-weighted (T2W), and DWI are commonly performed. Dynamic T1W contrast-enhanced sequence is still a foundation of any MRI program [11]. T1W sequences were performed before and after contrast administration to show and identify enhanced anomalies. After contrast administration, all lesions which are 5mm or larger were depicted as enhancing mass. T2W images allow better depiction of the lesion morphology and lesions with fluid (e.g., breast cysts) are better identified on this sequence.

Diffusion-Weighted Imaging (DWI) sequence

The random water molecular movement within tissue is influenced by the microstructure of tissue, and its cellular density is quantified by DWI. To achieve this, motion-sensitizing gradients (b factors) are used in the T2W echo-planar imaging (EPI) sequence. Because of the increased cell density in cancers, water diffusion is reduced, resulting in higher signal intensity during DWI [12].

Apparent Diffusion Coefficient (ADC) Sequence and its Corresponding Values

ADC is a quantitative measurement of diffusion derived from DWI. Values are expressed in 10^−3^ mm^2^/s. Because of the hampered diffusion in carcinomas, mean ADC is generally low (range: 0.8-1.3×10^−3^ mm^2^/s) compared with that of benign lesions (range: 1.2-2.0×10^−3^ mm^2^/s) [13].

Dynamic Evaluation with Time-Signal Intensity Curves

The permeability of vessels that will supply a lesion is investigated using dynamic analysis [11]. Benign and malignant masses have different enhancement patterns based on the type of lesion [14]. Benign lesions tend to have a continuous increase, whereas malignant lesions tend to have a reduction in the late phase. The most suspicious curve found "washout > plateau > persistent" inside a tiny region of interest (ROI) in the lesion is utilized to enhance lesion classification. A washout curve is present in approximately 85% of malignancies [11].

Kinetic Curves Types

There are three different types of kinetic curves. The kinetic curve assessment involves two phases - the initial phase and the delayed phase. The initial/early enhancement phase (two minutes after agent injection) can be classified as a slow, medium, or rapid increase in the curve. Rapid enhancement is characterized as >90% increase in initial peak signal strength within 90 seconds, which is highly predictive of malignancy. The "delayed phase" is defined as the signal strength two minutes after contrast injection, which is categorized into "persistent" (type I), "plateau" (type II), and "washout" (type III). The first rise usually indicates the extent of tumor angiogenesis, whereas the subsequent rise usually reflects the extent of tumor angiogenesis [15].

MR Spectroscopy

It is a non-invasive diagnostic technique that measures chemical information from a specific location within a tissue [16]. The spectra generated by MRS represent all observable metabolites in the region of interest, along with their individual chemical profiles. Different chemical compounds, such as phosphoethanolamine, choline, phosphocholine, and glycerophosphocholine (the latter three together are simply referred to as total choline [tCho]), and non-choline compounds, have been ascribed to the presence of a compound resonance about 3.23 ppm [17].

In the diagnostic situation, magnetic resonance spectroscopy (MRS) is now used to distinguish malignant from benign lesions based on higher tCho levels in malignant lesions, which have been linked to increased cellular membrane turnover [18].

Breast lesion evaluation on MRI breast

The American College of Radiology Breast Imaging Reporting and Data System standardizes breast MRI reporting (BI-RADS) using its lexicon [19]. The morphology and enhancement kinetics descriptors in the ACR BI-RADS MRI lexicon are the two major types of descriptors in describing a breast pathology. Initially, the breast composition and the quantity of background parenchymal enhancement (BPE) are assessed. A higher proportion of it is related to a higher chance of malignant etiology. A higher fraction of the BPE will usually lead to a higher risk of false-positive findings. Focuses, masses, and non-mass enhancement (NME) are the three types of lesions. The shape, borders, and internal enhancing pattern of masses are also used to classify them [11].

Approximately two-thirds of cancers will manifest as mass lesions, including invasive ductal cancers [20]; typical malignant tumors have an uneven size and/or margin, show washout, and will have heterogeneous/rim enhancing patterns. Based on the shape, mass is divided into round, oval, lobulated, or irregular. The edge or margins of the lesion are classified as smooth, uneven, spiculated. Interior mass enhancement can be either homogeneous, heterogeneous, rim enhancement, or dark internal septations. Other symptoms such as lymphadenopathy and pectoralis muscle invasion were also described [21].

Role of MRI in assessing metastasis to axillary lymph nodes

The identification of lymph node metastases has a substantial impact on the staging, therapy, and prognosis of patients with initial breast cancer. Preoperative imaging of the axilla and sampling of suspicious lymph nodes are critical tasks for the radiologist. The goal is to evaluate and detect the existence of the metastatic disease in non-palpable axillary lymph nodes (low or high tumor load) with a high enough positive predictive value to select patients for axillary lymph node dissection upfront [22].

Role of DWI and ADC values in the assessment of metastasis to axillary lymph nodes

In the differentiation of malignant and benign lymph nodes, measurement of ADC obtained from DWI added to conventional MR increases specificity and provides more accurate differentiation [23]. These lymph nodes are assessed the same as breast lesions on DWI and ADC sequences. Metastatic lymph nodes show restricted diffusion, and benign lymph nodes show no restricted diffusion. In the study conducted by Kim et al., the ADC value for metastatic lymph nodes was 0.91 × 10^-3^ mm^2^/s, and it was 1.27 × 10^-3^ mm^2^/s for benign lymph nodes [24].

## Materials and methods

Source of data

The study was a prospective observational study conducted on 41 patients with 54 breast lumps, which were diagnosed clinically and/or who underwent sonomammography or X-ray mammography at the Department of Radiodiagnosis at R.L. Jalappa Hospital and Research Center attached to Sri Devaraj Urs Medical College (SDUMC), Kolar. Prior informed consent was taken from the patients for their willingness to participate in the study. Patients who had undergone fine needle aspiration cytology (FNAC) or biopsy within three weeks, patients with recurrent breast cancer, and those who underwent chemotherapy or radiotherapy of the breast were excluded from the study.

Method of collection of data

**Figure 1:**
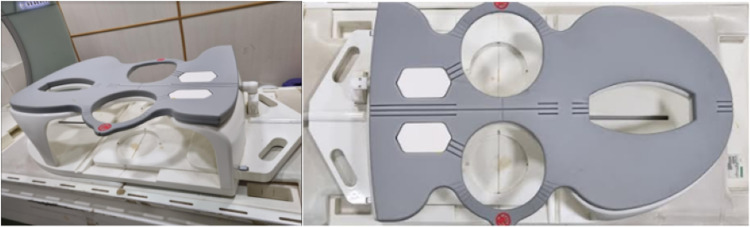
Dedicated double breast coil

Baseline data of the patients participating in the study were recorded. MR mammography was performed on 1.5 Tesla, 18 channel, MR Scanner (MAGNETOM® Avanto, Siemens AG, Munich Germany) using a dedicated double breast coil (Figure 1).

To avoid motion artefacts while performing MR mammography, cushions of varying sizes were used to hold breasts firmly. The patients were made to lie down in a prone position, and the following sequences were performed: T1 fat saturated (FS) and T2 axial images, DCE study and kinetic curves, DWI at 50, 400, and 800 s/mm^2^ b values with corresponding ADC values, and MR spectroscopy.

Before the delivery of contrast material, a native T1W FS sequence was obtained. The contrast was given at a max dose of 0.1 mmol/kg body weight. The bolus (nearly 20 mL) of contrast was flushed with normal saline before use. Three separate T1 FS contrast enhancement sequences of the equal number of sections as that of the plain study were acquired within a total time of five to seven minutes. Enhancement of the lesion after contrast administration was assessed. Most breast tumors showed a peak enhancement within two minutes following contrast material injection. Kinetic curves were derived, and their pattern of enhancement on initial and late phases was assessed. Breast masses were assessed as described in Table 1. 

**Table 1:** Parameters in the assessment of breast and breast mass on MR mammography

Assessment of breast tissue	
Breast composition	Amount of fibroglandular tissue A) almost entirely fat; B) scattered fibroglandular tissue; C) heterogeneous fibroglandular tissue; D) extreme fibroglandular tissue
Background parenchymal enhancement	Minimal mild-moderate marked
Assessment of breast mass	
Shape	Oval (includes lobulated), round, irregular
Margin	Circumscribed, not circumscribed, irregular, spiculated
Internal enhancement characteristics	Now under masses: internal enhancement characteristics A) homogeneous, B) heterogeneous enhancement, C) rim enhancement, D) dark internal septations
Kinetic curve assessment	Type I; Type II; Type III

Next, DWI sequences at 50, 400, and 800 s/mm^2^ b values were taken, followed by their corresponding ADC sequence. Both the sequences, i.e., DWI at 800 s/mm^2^ b value and ADC sequence, were compared to assess the presence or absence of restricted diffusion within the breast mass. For deriving ADC values, as per Hirano et al. [25], a feasible size of each region of interest (ROI) measuring ~25 mm^2^ was chosen to be placed over the lesions. In the case of malignant lesions, multiple oval-shaped regions of interest each measuring ~25 mm^2,^ were drawn over the areas of restricted diffusion. The value of each ROI was measured, and the mean of all the ROIs was taken as the final ADC value of the breast mass. In the case of benign breast masses, multiple ROIs each measuring ~25 mm^2,^ are drawn throughout the lesions as the lesions show no restricted diffusion. The mean of all the ROIs is taken as the final ADC value for the benign lesion. Proton MR spectroscopy is derived over the lesion, and the presence or absence of the choline peak was assessed and tabulated.

Axillae of all the patients were assessed for any enlarged lymph nodes. These lymph nodes were compared on DWI sequence of 800 s/mm^2^ b value with that of ADC sequence. Restricted diffusion of these lymph nodes was assessed, and for calculating its corresponding ADC value, a single oval-shaped ROI, which almost includes the entire axillary lymph node, was drawn, and the value derived is taken as the ADC value of the lymph node.

The data were entered in a Microsoft Excel sheet. The measurable variables were analyzed and interpreted between them by the Student's t-test, and the ordinal and categorical variables between them were interpreted by Chi-square (χ^2^) test. The predictive value of mpMRI for differentiating benign and malignant lesions was estimated. The statistical procedures were performed with the help of an SPSS statistical package (version 21; IBM Inc., Armonk, USA) and OpenEpi (version 3.01). A p-value less than 0.05 was considered statistically significant.

## Results

In this study, 54 breast lesions from 41 patients were assessed. Among the study population, 40 (97.6%) participants were female, and only one (2.44%) participant was male. Most of the patients were in the perimenopausal age group of 40-59 years (n=14; 34.1%), followed by 50-59 years (n=13; 31.7%; see Table 2).

**Table 2:** Age group distribution of the patients included

Age group (in years)	Number of patients
< 20	2
20-29	3
30-39	7
40-49	12
50-59	9
60-69	5
> 70	3

The mean age of the patients was 47.1±14.7 years (mean±SD), with a range of 16 to 75 years. Out of 41 participants, 32 patients were reported to have only one breast lesion; the rest of the patients had more than one breast lesion in the same breast or in the contralateral breast.

Assessment of the breast tissue and the breast mass is based on the ACR-BIRADS MRI lexicon. 

Descriptors - modifiers describing breast tissue

Breast tissue was assessed for fibroglandular tissue on the plain study and the amount of background parenchymal enhancement (BPE) in the post-contrast study [21].

1. Breast tissue - fibroglandular tissue: out of the four categories of breast composition, 12 patients were reported to have scattered fibroglandular tissue and heterogenous fibroglandular tissue each; 11 patients had almost entirely fat content within the breast with minimal or absent fibroglandular tissue and six had breast with extreme fibroglandular tissue within. 

2. Breast tissue - background parenchymal enhancement (BPE): based on the amount of enhancement of the fibroglandular tissue of the breast after contrast administration, BPE is broadly divided into four categories of minimal, mild, moderate, or marked enhancement of the breast tissue. We observed that most of the patients had minimal or mild BPE (Table 3).

**Table 3:** Descriptive analysis of type of breast composition and background parenchymal enhancement (BPE) in the study population (n=41)

Parameters of breast tissue assessment	Number of patients
Breast composition	
Almost entirely fat	11
Scattered fibroglandular tissue	12
Heterogenous fibroglandular tissue	12
Extreme fibroglandular tissue	6
Background parenchymal enhancement (BPE)	
Minimal	13
Mild	13
Moderate	11
Marked	4

Descriptors - modifiers describing a mass

Following identification of the breast mass on MRM, shape, margins, enhancement pattern, and kinetic curves of the breast masses were assessed.

**Figure 2:**
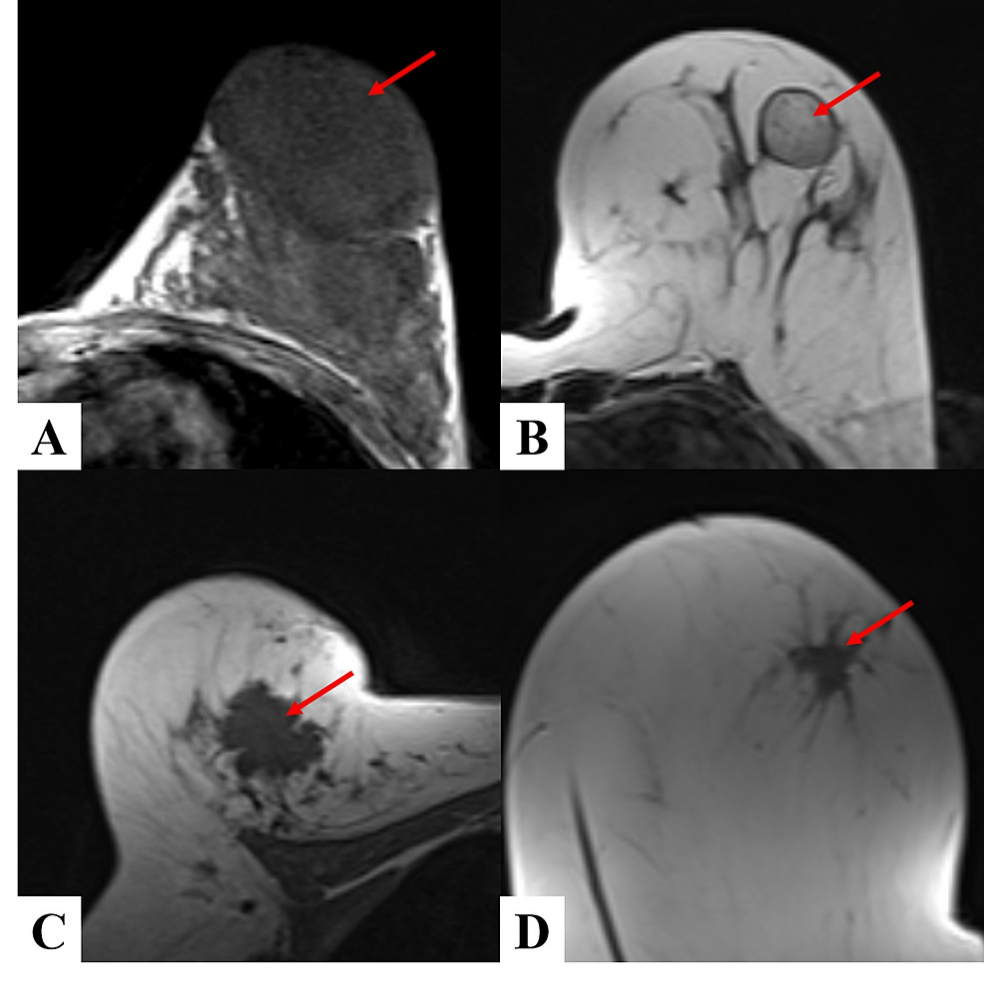
Shape of breast lesions (A) oval, (B) round, (C) lobulated, (D) spiculated

1. Shape of the lesion: as per morphology of the enhancement breast lesion studied, out of all the 54 lesions assessed, 15 lesions were irregular in shape, 20 were oval in shape, and the remaining 19 lesions were round (Figure 2 and Table 4-A) 

**Table 4:** Descriptive analysis of breast mass

	Descriptors – modifiers describing a mass	Number of mass
A	Shape: describes the overall morphology of the enhancement	
	Irregular	15
	Oval	20
	Round	19
B	Margin: describes the borders	
	Circumscribed	33
	Irregular	16
	Spiculated	5
C	Enhancement pattern	
	Non-mass enhancement (NME)	4
	Homogeneous	11
	Heterogeneous	24
	Rim enhancement	1
	Dark internal septations	14
D	Kinetic curves	
	Type I	25
	Type II	17
	Type III	12

**Figure 3:**
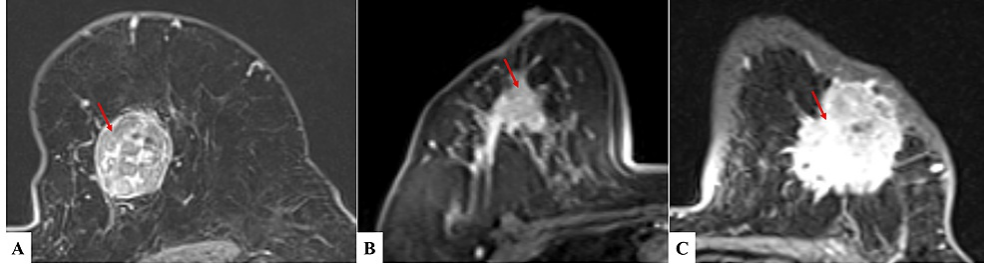
Margin of breast lesion on T1 weighted post-contrast images (A) circumscribed, (B) irregular, (C) spiculated

**Figure 4:**
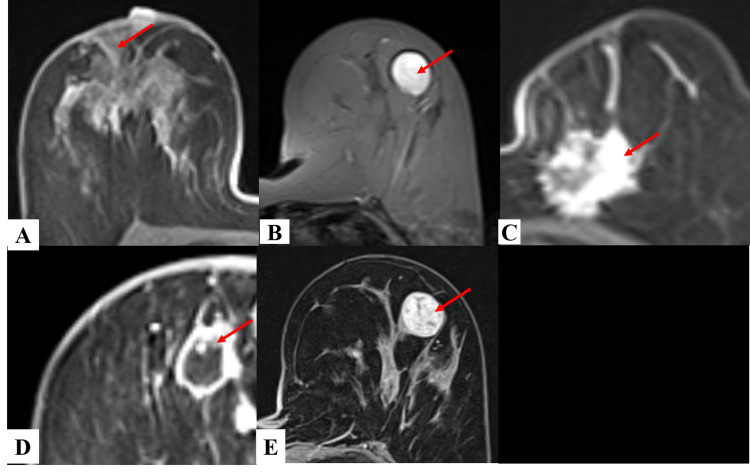
Five patterns of post-contrast enhancement of breast lesions on contrast-enhanced T1 fat-saturated MR images (A) non-mass-like enhancement, (B) homogenous enhancement, (C) heterogeneous enhancement, (D) rim enhancement, (E) enhancement of the lesion with dark internal septations

**Figure 5:**
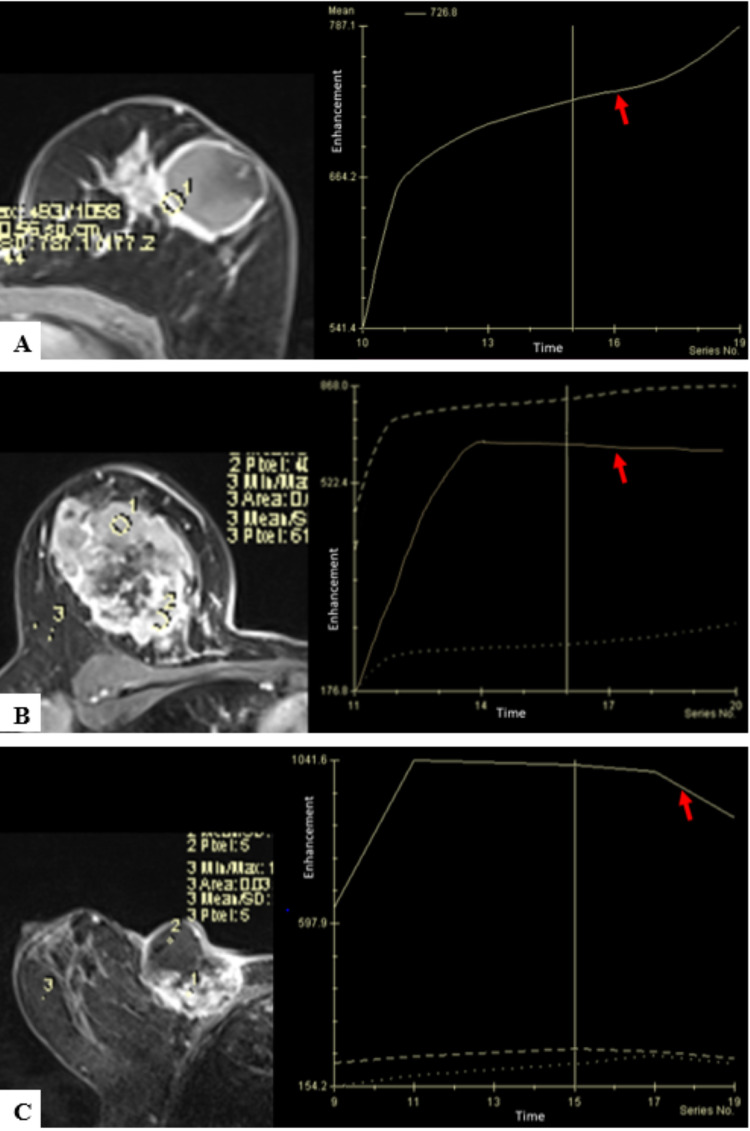
Kinetic curve assessment according to types (A) Fibroadenoma in left breast showing type I kinetic enhancement curve. (B) Infiltrative ductal carcinoma in right breast showing type II kinetic enhancement curve. (C) Squamous cell carcinoma in right breast showing type III kinetic enhancement curve.

2. Margins of the lesion: it describes the border and extent of the lesion. The lesions were broadly divided as circumscribed or not circumscribed. The lesions which were not circumscribed were subdivided into two categories - lesions with irregular margins or spiculated margins. As we observed, most of them, i.e., 33 lesions (61.11%), were circumscribed. Twenty-one lesions had margins, which were not circumscribed, out of which 16 (29.63%) lesions had irregular margins, and the rest five (9.26%) were found to have spiculated borders (Figure 3, Table 4-B). 

3. Internal enhancement characteristics: lesions were identified to enhancement either as a mass or as a non-mass-like enhancement. All the masses were observed to have either of the four types of enhancement - homogenous, heterogeneous, rim enhancement or enhancement with dark internal septations. In our study, the majority of the mass (24, 44.44%) showed the heterogeneous type of enhancement, followed by enhancement with dark internal septations in 14 (25.93%) masses, and homogeneous type of enhancement was seen in 11 (20.37%) lesions. Four of the lesions showed non-mass-like enhancement constituting 7.4% of the total study population (Figure 4, Table 4-C). 

4. Kinetic curve assessment: enhancement curves of the breast lesions following contrast administration was divided into type I, II, or III following assessment of the signal intensity/time curve on both initial and delayed phase. As per lesions studied, 25 (46.30%) were found to show type I enhancement curve, 17 (31.48%) of them had type II and 12 (22.22%) had type III kinetic curves (Figure 5, Table 4-D).

Diffusion-weighted imaging and apparent diffusion coefficient​​​​​​​

**Figure 6:**
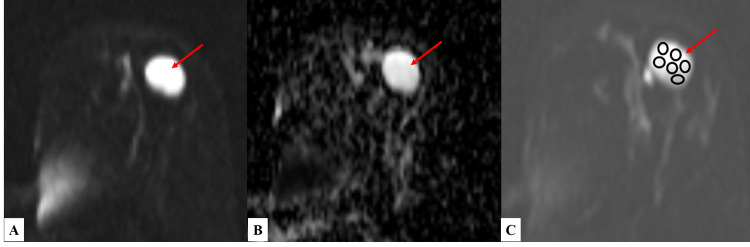
DWI and ADC sequences of a patient with fibroadenoma (A) DWI and (B) corresponding ADC images showing no restriction of diffusion within the lesion present in the left breast – suggestive of benign etiology. (C) DWI image demonstrating the method of placing multiple ovoid ROIs (each measuring ~25 mm^2^) throughout the benign lesion for calculation of mean ADC value. DWI - diffusion-weighted imaging; ADC - apparent diffusion coefficient; ROI - region of interest

**Figure 7:**
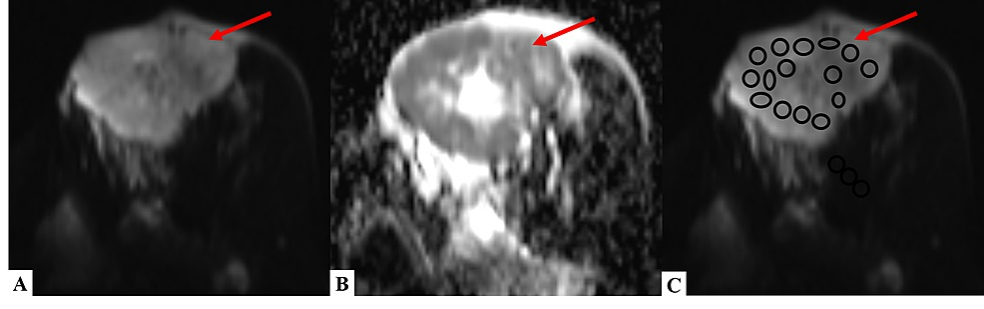
DWI and ADC sequences of a patient with infiltrative ductal carcinoma (A) DWI and (B) corresponding ADC images showing the peripheral area of restriction of diffusion within the lesion present in the left breast- suggestive of malignant etiology. (C) DWI image demonstrating the method of placing multiple ovoid ROIs (each measuring ~25 mm^2^) in the areas of restricted diffusion for calculation of mean ADC value. DWI - diffusion-weighted imaging; ADC - apparent diffusion coefficient; ROI - region of interest

All the lesions were assessed for restricted diffusion, if present or not, by comparing the DWI sequence at 800s/mm^2^ with its corresponding ADC sequence. Out of the 54 lesions, 29 (53.7%) showed restricted diffusion, and all were diagnosed to be malignant mass on histopathology. Twenty-five (46.3%) lesions showed no restricted diffusion. Twenty of them were confirmed to be benign lesions, but five lesions on histopathological examination (HPE) were diagnosed to be malignant (Table 5). Apparent diffusion coefficient values of all the breast masses were calculated. Thirty (55.56%) lesions had ADC value <1.3 × 10^−3^ mm^2^/s and 24 (44.44%) had ADC value >1.3 × 10^−3^ mm^2^/s (Figures 6-7). 

**Table 5:** Findings on DWI sequence and ADC values of breast lesions (n=54) DWI - diffusion-weighted imaging; ADC - apparent diffusion coefficient

Restricted diffusion on DWI	Number of mass
Present	29
Absent	25
ADC values (× 10^−3^ mm^2^/s)	Number of mass
ADC value <1.3	30
ADC value >1.3	24

MR Spectroscopy

**Figure 8:**
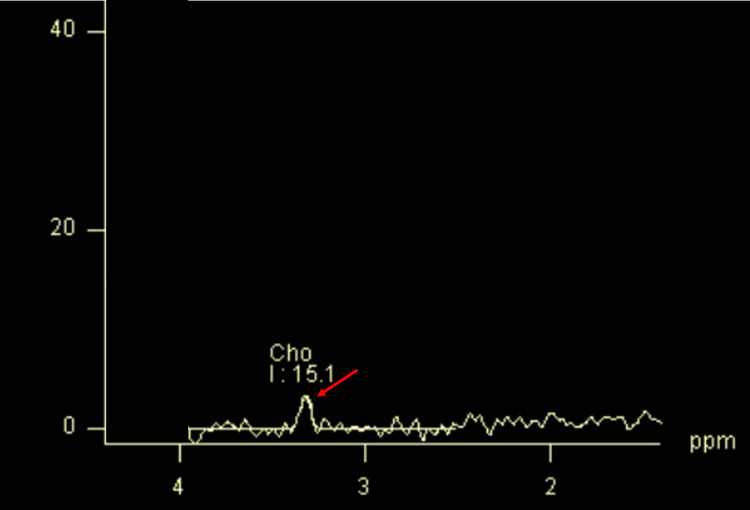
MR spectroscopy image of a malignant breast lesion showing tCho peak at 3.2 ppm tCho - total choline

As per MRS of the lesions studied, 28 (51.85%) showed a choline peak, and the rest 26 showed no choline peak (Figure 8, Table 6). 

**Table 6:** Descriptive analysis of choline peak on MR spectroscopy in the lesions studied (n=54)

MR spectroscopy - choline peak	Number of mass
Yes	28
No	26

Final diagnosis on MRM and its comparison with histopathological findings

As per MRI final diagnosis, 29 (53.70%) lesions were diagnosed as malignant and 25 (46.30%) as benign (Table 7). The histopathological type of the breast lesions was assessed, and the diagnoses were compared with MRM's final diagnoses as benign or malignant breast lesions. On histopathology, 34 lesions were diagnosed as malignant and 20 lesions as benign. Only male patient included in the study was diagnosed to have liponecrosis.

**Table 7:** MRI final diagnosis in the masses studied (n=54)

MRI final diagnosis	Number of mass	Percentages
Malignant	29	53.70%
Benign	25	46.30%

Fibroadenomas were the most common benign, and infiltrating ductal carcinoma was the most common malignant breast mass (Table 8).

**Table 8:** Histopathological diagnosis of the breast masses studied (n=54)

Histopathological diagnosis	Number of mass	Percentages
Fibroadenoma	18	33.33%
Infiltrating ductal carcinoma	17	31.48%
Pure mucinous carcinoma	5	9.26%
Ductal carcinoma	4	7.41%
Squamous cell carcinoma	3	5.56%
Benign phyllodes tumor	1	1.85%
Ductal carcinoma in situ	1	1.85%
Intracystic papillary carcinoma	1	1.85%
Liponecrosis	1	1.85%
Lobular carcinoma in situ	1	1.85%
Medullary carcinoma	1	1.85%
Secretory carcinoma	1	1.85%
TOTAL	54	100%

As per the HPE report, 34 (62.96%) lesions were diagnosed to be malignant, and 20 (37.04%) were benign in etiology (Table 9).

**Table 9:** Number of benign and malignant mass on HPE (n=54) HPE - histopathological examination

HPE	Frequency	Percentages
Malignant	34	62.96%
Benign	20	37.04%

Out of 34 malignant lesions on HPE, 29 lesions showed correlative findings of a malignant mass on both mpMRI and histopathology. But five masses that were diagnosed to be mucinous carcinomas on HPE showed false-negative features as a benign lesion on MRM, as they were well-circumscribed, homogenously enhancing lesions showing no restricted diffusion, high ADC value of >1.3 × 10^−3^ mm^2^/s and no tCho peak on MRS.

Out of 20 HPE confirmed benign breast masses, 19 masses showed all features of benign etiology on all the parameters of mpMRI. But only one breast mass had an ADC value of <1.3 × 10−3 mm2/s (finding consistent with malignant lesion) but all other mpMRI parameters were consistent with findings that of a benign breast mass. Rest 19 masses had ADC value >1.3 × 10^−3^ mm^2^/s. The difference in the proportion of ADC value between HPE status was statistically significant (p-value <0.001; Table 10). 

**Table 10:** Comparison of restricted diffusion on DWI and ADC values with HPE (n=54) DWI - diffusion-weighted imaging; ADC - apparent diffusion coefficient; HPE - histopathological examination

	HPE	
Parameters	Benign (n=20)	Malignant (n=34)
Restricted diffusion on DWI		
Present	0 (0%)	29 (85.29%)
Absent	20 (100%)	5 (14.71%)
ADC values		
ADC Value <1.3	1 (5%)	29 (85.29%)
ADC Value >1.3	19 (95%)	5 (14.71%)

Therefore, out of 20 benign lesions from HPE, all 20 were labeled as benign by MRI. Out of 34 malignant lesions from HPE, five were labeled as benign by MRI, and 29 (85.29%) were labeled as malignant by MRI (Table 11). 

**Table 11:** Comparison of MRI final diagnosis (malignant/benign) with HPE (n=54) HPE - histopathological examination

MRI final diagnosis	HPE	
	Benign (n=20)	Malignant (n=34)
Benign	20 (100%)	5 (14.71%)
Malignant	0 (0%)	29 (85.29%)

​​​​​​​Axillary lymph nodes​​​​​​​

**Figure 9:**
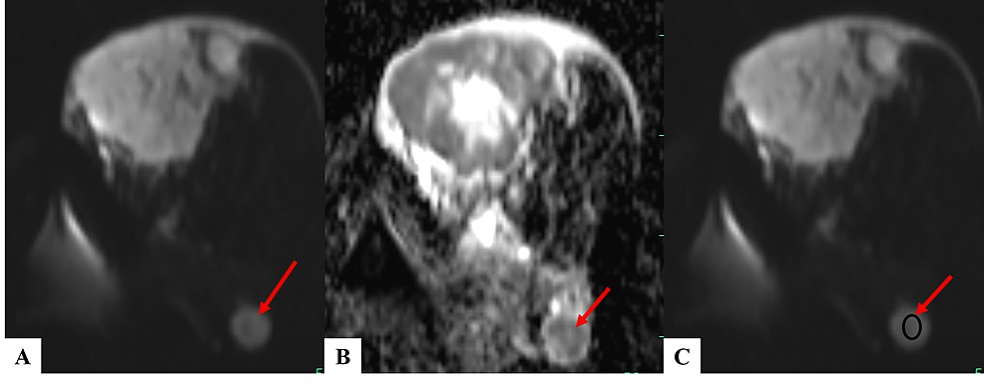
DWI and ADC sequences of the axillary lymph node in a patient with infiltrative ductal carcinoma in the left breast (A) DWI and (B) corresponding ADC images showing a lymph node in the left axilla with restriction of diffusion. (C) DWI image demonstrating method placing a single ovoid ROI on the lymph node for calculation of ADC value. DWI - diffusion-weighted imaging; ADC - apparent diffusion coefficient

Restricted diffusion of the 24 enlarged axillary lymph nodes was assessed, and its corresponding ADC values were calculated. As we observed, 16 (51.85%) axillary lymph nodes showed restricted diffusion, and they had an ADC value <1.4 × 10^−3^ mm^2^/s, the rest eight enlarged lymph nodes, which showed no restricted diffusion, had an ADC value of >1.4 × 10^−3^ mm^2^/s (Figure 9, Table 12). 

**Table 12:** Descriptive analysis of restricted diffusion in axillary lymph nodes (n=24) ADC - apparent diffusion coefficient

Axillary lymph nodes - restricted diffusion	Frequency	ADC values of the lymph nodes
Present	16	< 1.4 x 10^-3^
Absent	8	> 1.4 x 10^-3^

## Discussion

The DCE and DWI techniques of MRM are the most sensitive imaging technique used in detecting breast cancer. In recent years, these techniques have out-performed traditional ultrasonography and mammography. The DCE-MRI has several indications in diagnosing breast lesions, as it has good sensitivity and specificity for the detection of breast cancer [26]. We analyzed the role of the discrete BI-RADS descriptors for DCE-MRI (shape, margin, type of enhancement, internal enhancement pattern/characteristics, and enhancement kinetics) and restricted diffusion on DWI and its ADC values; and correlated these findings with the histopathology of the lesions. Very few studies [27] have developed mpMRI models in diagnosing breast cancer. These models have proven to be very valuable in the characterization of lesion and staging.

As per lesions studied, out of 54 lesions, 15 lesions had an irregular shape, 20 had an oval shape, and 19 had a round shape. The majority of the lesions (33) had circumscribed margins rest of the 21 lesions had non-circumscribed margins (16 irregular, five spiculated). Heterogenous lesions were found in the majority of the participants (44.44%), followed by enhancement with dark internal septations in 25.93% and homogeneous enhancement in 20.37%. In a study by Zhang et al. [27] on DCE-MRI, DCE morphological features associated with breast cancer presented as masses having irregular shape, irregular/spiculated margin, and heterogeneous/rim internal enhancement pattern (p<0.0001). The significant morphological features presenting in benign lesions were masses with round/oval shape, with circumscribed margins and dark/homogenous septations internal enhancement pattern (p<0.0001).

The kinetic curves studied among the study population found type I in 46.30%, type II in 31.48%, and type III in 22.22%. In a study by Zhang et al. [27], when they included the kinetic curves in their mpMRI, they found that lesions with plateau or washout kinetic curves had a 3.7-fold risk of being malignant than lesions with persistent enhancement.

Restricted diffusion on DWI was found in 29 breast masses, and all these lesions which showed restricted diffusion had an ADC value of <1.3 ×10^-3^ mm^2^/s. All other 25 lesions showed no restricted diffusion; 19 of these lesions had an ADC value of >1.3 ×10^-3^ mm^2^/s, one non-restricting lesion showed a value of 1.2 ×10^-3^ mm^2^/s and was diagnosed to be benign phyllodes tumor on histopathology. The remaining five lesions also showed no restricted diffusion and had an ADC value of >1.3 ×10^-3^ mm^2^/s, but these five lesions were diagnosed to be mucinous carcinomas on histopathology. The difference in the proportion of ADC value between HPE status was statistically significant (p-value <0.001). ADC values showed a specificity of 95% in diagnosing breast mass.

All the malignant lesions showed increased tCho peak on MRS, and benign lesions showed no peak. From the past literature, it is found the presence of a tCho peak to be a reliable marker for the detection of malignancy [28].

Few of the benign lesions that can be misdiagnosed on MRM to be malignant are papillomas, adenosis, atypical hyperplasia, and benign phyllodes tumor. And similarly, mucinous carcinoma is one of the malignant conditions which can be misdiagnosed to be harmless as these lesions show few of the characteristics of benign lesions on MRM like smooth margins, no restricted diffusion, and sometimes these lesions do not enhance [29]. In our study, we observed that mucinous carcinomas were falsely misdiagnosed as benign breast mass; and on the other side, though benign phyllodes tumor was diagnosed to be benign only on ADC, it showed a value < 1.3 × 10^−3^ mm^2^/s.

On MRM, the lesions were divided into benign and malignant based on the features as described below.

Benign breast lesions: the lesions which were well-defined, round or ovoid in shape with smooth margins; showing no enhancement or homogenous enhancement or enhancement with dark internal septations; type I kinetic curve on post-contrast study; no restricted diffusion with an ADC value of >1.3 ×10^-3^ mm^2^/s and no tCho peak on MRS were considered as benign.

Malignant breast lesions on MR mammography: the lesions which had irregular or spiculated margins, showing restricted diffusion on DWI with an ADC value of <1.3 ×10^-3^ mm^2^/s; displaying heterogeneous or rim enhancement on the post-contrast study with type II or III kinetic enhancement curves and those which showed choline peak on MR spectroscopy were diagnosed to be malignant.

A study by Naranjo et al. [26] found that their model constructed on mpMRI inclined to have the best diagnostic accuracy of 81.7% at the area under the curve (AUC) of 0.85. Additionally, identifying the area having maximum restricted diffusion within the breast mass on DWI with ADC mapping helps in targeting/selecting the most appropriate site for biopsy, as it depicts the most aggressive site of lesion and hence diminishing an error in sampling [27].

The study by Razek et al. [30] found the mean ADC value in metastatic axillary lymph node to be 1.08 ± 0.21×10^−3^ mm^2^/s and in benign lymph nodes was 1.58 ± 0.14 × 10^−3^ mm^2^/s. In our study, we observed that all the lymph nodes which showed restricted diffusion on DWI and had an ADC value of <1.4 × 10^−3^ mm^2^/s were considered as malignant/metastatic lymph nodes. Lymph nodes, which showed no restricted diffusion and had an ADC value of >1.4 × 10^−3^ mm^2^/s, were considered as benign lymph nodes.

Limitations 

The present single-center study was performed on a relatively small study population. Increasing the sample size would improve the statistical power of the results. This study included only breast mass and did not evaluate the diagnostic performance in diffuse inflammatory/infective conditions such as mastitis. Multicentric studies involving larger groups of patients are needed for evaluating the feasibility and in further improving the diagnostic accuracy and specificity of MRM.

## Conclusions

Breast cancer is the leading cause of mortality and morbidity among women. Early screening of breast lesions becomes important to determine a good prognosis. Ultrasound mammography is the most widely accepted and traditional tool used in screening breast lesions. At present, DCE-MRM, along with advanced techniques such as DWI, ADC values, and MRS, makes a more appropriate and precise diagnosis of breast lesions. Hence the present study aimed to assess the morphology of breast mass using mpMRM along with DWI, ADC values, and tCho peak on MRS. This study shows that mpMRM assessment of breast masses, which included DCE-MRI, DWI, ADC values, and MR spectroscopy, had higher sensitivity of 85.2% and specificity of 100%. This leads to better non-invasive accurate diagnosis of the masses and hence helps in better treatment to the patient.
